# Patterns of eating behaviors, physical activity, and lifestyle modifications among Bangladeshi adolescents during the COVID-19 pandemic

**DOI:** 10.1371/journal.pone.0302571

**Published:** 2024-05-09

**Authors:** Most. Zannatul Ferdous, Md. Saiful Islam, Lakshmi Rani Kundu, Ummay Soumayia Islam, Rajon Banik, Shahina Pardhan

**Affiliations:** 1 Department of Public Health and Informatics, Jahangirnagar University, Savar, Dhaka, Bangladesh; 2 Centre for Advanced Research Excellence in Public Health, Savar, Dhaka, Bangladesh; 3 Vision and Eye Research Institute, School of Medicine, Anglia Ruskin University, Cambridge, United Kingdom; United Arab Emirates University, UNITED ARAB EMIRATES

## Abstract

**Background:**

Several safety measures like movement restrictions, closure of educational institutions, and social distancing measures continue over the world including Bangladesh during the COVID-19 pandemic. This study aimed to examine the patterns of eating behaviors, physical activity, and lifestyle modifications among adolescents during the COVID-19 pandemic residing in Bangladesh.

**Methods:**

A cross-sectional study was performed among 490 adolescents in Bangladesh from December 2020 to May 2021. The survey was carried out through a semi-structured web-based questionnaire that asked questions about socio-demographics (i.e., age, sex, marital status, education, residence), perceived health status and quality of life, anthropometrics (i.e., height, weight), dietary habits (i.e., frequency of eating, daily intake of certain foods, number of meals eaten daily), and physical activity (i.e., modified version of the International Physical Activity Questionnaire Short Form [IPAQ-SF]), as well as, pre- and during COVID-19 information on stress, and sleep.

**Results:**

During the pandemic, 43.7% participants reported weight gain; and 23.5% reported an increased number of meals per day during COVID-19. Additionally, the participants’ eating habits diverged from the local balanced diet principles and were more akin to ‘unhealthy’ eating patterns. Though, during the COVID-19 pandemic, physical exercise slightly increased (>3 times/ week: 8.2% vs. 13.5%; *p*<0.001) compared to pre-COVID-19 period, the screen time for entertainment increased drastically (>5 hours/ week: 12.2% vs. 27.3%; *p*<0.001). Compared to the pre-pandemic, a sizeable proportion of individuals experienced more physical tiredness, emotional exhaustion, irritation, and stress (*p*<0.001) during the pandemic. During the pandemic, 47.5% of participants experienced different sleep difficulties.

**Conclusions:**

Although lockdowns and social distancing are important safety measures to protect people from COVID-19, findings reveal that they might cause a variety of lifestyle changes, physical inactivity, and psychological issues in Bangladeshi adolescents.

## Introduction

An unparallel influence on lifestyle, societies, and human health has been caused by the novel coronavirus disease (COVID-19) pandemic, which has had far-reaching consequences [1, 2]. The COVID-19 pandemic has spread since it was first identified in Wuhan, Hubei Province, China, at the end of 2019 and has become a serious public health emergency globally [3, 4]. More than 122 million individuals worldwide have been impacted by COVID-19, which the World Health Organization (WHO) has classified as a pandemic [5, 6]. Countries around the world have enacted exceptional restricted measures, including full or partial lockdowns, isolation, quarantine, and social distancing, to end the rapid spread of COVID-19 [7–9]. While these restrictions help lessen infection rates, these have led to significant changes in lifestyle patterns, particularly in terms of dietary habits and physical activity [10–12].

Consequent to food supplier enclosures, COVID-19 has disrupted regular eating patterns, perhaps leading to the consumption of unhealthy diets such as convenience meals, ultra-processed foods, and salty snacks [1, 2, 11]. Maintaining a perfect nutrition status is important, particularly in the time when a healthy immune system is crucial [12]. The WHO has advised that a healthy lifestyle (e.g., healthy diet, staying physically active, etc.) can help to fight against COVID-19 [13]. But prior study suggests that there were trends toward unhealthy eating behaviors, such as taking more calories, snacking more frequently, consuming less fresh fruits and vegetables, and gaining weight during lockdowns [14, 15]. Additionally, emotional and psychological reactions to the COVID-19 epidemic [16, 17], may raise the chance of abnormal eating habits including eating behaviors such as overeating, specifically ‘comfort foods’ rich in sugar, which are referred to as ‘food carvings’ [18, 19]. Quarantine-induced anxiety and boredom are considered determinants for eating more food and food of lower quality when contrast to normal living conditions [11].

Restrictions connected to COVID-19 drastically decreased physical activity, sport, and exercise, resulting in vicious cycles that compromise physical fitness while increasing sedentary behavior [10, 14, 20]. Physical inactivity is one of the most important predictors of morbidity around the world [21]. This is true not only for the general population, but also for elderly people and individuals with chronic illnesses, who are at higher risk because of COVID-19 [22]. Also, being physically active is linked favorably to having the capacity to deal with infections as well as the immunologic and a cardiac problems of more serious results [15, 23]. According to a recent survey, 38.5% of UAE residents do not engage in physical activity and 36.2% spend more than 5 hours per day watching screens for refreshments [1]. Another study among Latin Americans also found a decline in physical activity during the COVID-19 pandemic [2]. The substitution of ultra-processed meals for handmade and fresh foods increases obesity, non-communicable diseases (NCDs), and specific nutritional deficiencies throughout all life stages, particularly in adolescents [2]. Physical exercise and school-related activities are tightly connected in teenagers [24]. Physical activity has been restricted due to the COVID-19 pandemic-related shutdown of schools, potentially raising the risk of long-term sedentary behavior.

Considering the existing COVID-19 pandemic, it is vital to examine how adolescent physical activity and lifestyle, as well as dietary patterns, have changed, as these factors could be future risk factors for NCDs. In Bangladesh, there is a scarcity of evidence to explore the impact of COVID-19 on eating behaviors and lifestyle patterns, particularly among adolescents. Consequently, the study sought to determine how adolescents in Bangladesh changed their dietary patterns, level of physical activity, and lifestyles before amid during the COVID-19 pandemic.

## Methods

### Study design and population

A cross-sectional study was conducted to identify the patterns of eating behaviors, physical activity, and lifestyle modifications among 490 Bangladeshi adolescents (50.6% males; mean age: 16.3 ± 2.4 years; age range: 10–19 years) during the COVID-19 pandemic from December 2020 to May 2021. The inclusion criteria were: ⅰ) being an adolescent (age range: 10–19 years), ⅱ) being a Bangladeshi resident, and ⅲ) having a willingness to participate. Responses from participants outside the age range and incomplete surveys were excluded from the study.

### Sample size

The sample size was calculated using the following equation:

$$n=\frac{z^{2}\times p\times (1-p)}{d^{2}}$$

$$n=\frac{1.96^{2}\times 0.5\times (1-0.5)}{0.05^{2}}$$

=384.16

≈384

Where, n = number of samples; *z* = 1.96 (95% confidence level); *p* = prevalence estimate (0.5); *q* = (1−*p*); *d* = precision limit or proportion of sampling error (0.05). Considering a 10% non-response rate, a total of 423.5 ≈ 424, the sample size was determined. This estimate exceeded our sample size.

### Study procedure

Research instrument tool for example a questionnaire (semi-structured) was used to conduct the survey. Informed consent along with all questions were incorporated in the Google survey tool (Google Forms). After that a link was created to circulate the survey. Various social media platforms (e.g., Facebook, WhatsApp, etc.) involving adolescents were utilized for sharing the link. In addition, participants were invited using a snowballing approach from close networks of members of the current study.

### Ethics

According to the Helsinki Declaration, all the procedures were performed with the principle of human investigations. Ethical permission was adopted from the Biosafety, Biosecurity and Ethical Committee of Jahangirnagar University [Ref: BBEC, JU/M 2021/COVID-19/11 (1)]. Data collection was completely anonymous and no personal data was included. Also, participants were not offered any kind of economic incentive.

### Data collection tools

A semi-structured questionnaire was used to collect data from participants which was adopted through extensive literature review [1, 25–27]. The questionnaire was translated using the most commonly used guideline [28]. This questionnaire was translated into the local language of the participants (in Bengali) and then, the backward translation was done (in English). Before starting the final survey, the piloting was conducted (with 15 individuals) to ensure the acceptability and clarity of the questionnaire among the participants. Minor changes were amended after the pilot testing (S1 File).

#### Socio-demographic information

Participants’ socio-demographic information was collected using a total of 10-item questions including sex (male/ female), age, marital status (unmarried/ married/ in a relationship), education level (secondary/ higher secondary), height, weight, weight change (decrease/ no change/ unaware), perceived health status (good/ poor), perceived quality of life (good/ poor), and residence (urban/ rural). Perceived health status and quality of life were assessed using two questions with a five-point Likert scale ranging from 1 (“poor”) to 5 (“excellent”) (*“In general*, *how would you rate your overall health*?*”* and *“In general*, *how would you rate your overall quality of life*?*”*), as used previously [29, 30]. A dichotomous form [good (excellent/ very good/ good) and fair/ poor (poor/ fair)] was used to present the responses [29].

#### Dietary assessment

The dietary assessment included dietary questions and categories of food comprised of the habitual intakes of the adolescent in Bangladesh [31, 32]. The questionnaire consisted of the following food groups: fruits, vegetables, milk and milk products, meat and meat products, grain (bread, rice, and pasta), sweets and sweetened beverages, coffee, tea, and energy drinks. The response options of this question contained: “never”, “1–4 times per week”, “2–3 times a day”, “4 or more times a day”. Internal consistency was assessed using Cronbach’s alpha for this section to reduce false high internal consistency and the value was 0.84.

In addition, other information on eating habits including meal type, meal frequency, eating breakfast, skipping meals, reasons for skipping meals, and water intake were also recorded from participants.

#### Physical activity assessment

Following a previous study [1], a modified version of the International Physical Activity Questionnaire Short Form (IPAQ-SF) was employed to assess physical activity both before and during the COVID-19 pandemic among the participants surveyed [26]. Concerning before and during the COVID-19 pandemic, participants were asked how many days per week they performed household chores, and how many days per week they engaged in moderate to intense physical activity. Regarding before and during the COVID-19 pandemic, they were also questioned *‘How many hours per day did they spend on the computer for work or study purpose*?*’* and *‘How many hours per day did they spend on screens for amusement and refreshment*?*’*.

#### Stress, irritability, and sleep assessment

Minor changes were made to the Copenhagen Psychosocial Questionnaire (COPSOQ-II) to provide questions about stress and sleep [30]. Before and during the COVID-19 epidemic, participants were questioned about how frequently they felt emotionally exhausted, physically exhausted, tense, and irritable with four possible responses (i.e., a large part of the time, part of the time, a small part of the time, and not at all). Participants were asked whether they experienced sleep disturbances regularly including sleeping badly and restlessly, having difficulty going to sleep, early wake-ups, and trouble back to sleep or none of the options. The response choices for rating sleep quality were “very good”, “good”, or “poor”. Two questions were asked two times: first regarding the time before COVID-19 and again during COVID-19.

### Statistical analysis

Descriptive statistics for example frequency and proportion were for categorical variables. For categorical variables, the Chi-square test was adopted to measure the relationship. The diversity of categorical variables before and during the COVID-19 pandemic was identified using the McNemar test. Group-related dietary practice into components was performed using Principal component analysis (PCA). Component loadings were utilized to determine the association between each food group and its underlying component. Values greater than 0.3 were taken into account in this research as having an impact on component construction. Based on the total component loadings of all the food groups, a score was assigned to each participant. Varimax rotation was used to the detected components to obtain orthogonal, uncorrelated factors and reduce variance errors. PCA’s suitability was evaluated using the Kaiser-Meyer-Olkin (KMO) sample adequacy measure. Findings were significant for *p*-values less than 0.05. Statistical package for the Social Sciences (SPSS) version 26.0 (IBM, Chicago, IL, USA) was taken to perform the analysis.

## Results

### Demographic characteristics

The survey was conducted among 490 participants with a mean age of 16.3 years (SD: 2.4; age range: 10–19 years). Table 1 depicts the general characteristics of the study participants. The majority of respondents were male (50.6%), unmarried (87.1%), had a higher secondary education (53.1%), and lived in urban areas (59.4%). The average height and weight of the participants were 62.3 inches and 55 kg respectively. 73.9% reported good health status and good quality of life (70.4%). However, the study revealed the proportion of weight gain was 43.7%, 12.4% reported weight loss, 29.8% managed their weight, and 14.1% were not aware of their weight. More than one-third of the participants (36.7%) maintained hunger and satiety changes during the pandemic, while 35.5% reported an increase, 15.5% reported a decrease, and 12.2% were unaware.

**Table 1:** Distribution of general characteristics (during the study periods).

Variables	Sub-categories	n	(%)
**Sex**	Male	248	(50.6)
	Female	242	(49.4)
**Marital status**	Unmarried	427	(87.1)
	Married	17	(3.5)
	In a relationship	46	(9.4)
**Academic grade**	Secondary	230	(46.9)
	Higher secondary	260	(53.1)
**Residence**	Urban	291	(59.4)
	Rural	199	(40.6)
**Self-reported health status**	Good	362	(73.9)
	Poor	128	(26.1)
**Self-reported quality of life**	Good	345	(70.4)
	Poor	145	(29.6)
**Weight changes during the pandemic**	Increase	214	(43.7)
	Decrease	61	(12.4)
	No change	146	(29.8)
	Unaware	69	(14.1)
**Sense of hunger and satiety changes during the pandemic**	Increase	174	(35.5)
	Decrease	76	(15.5)
	No change	180	(36.7)
	Unaware	60	(12.2)
**Continuous variables**		**Mean**	**(SD)**
**Age (year)**		16.3	(2.4)
**Height (inch)**		62.3	(9.5)
**Weight (kg)**		55.0	(11.2)

### Eating habits

Table 2 provides information about eating habits among the study participants before and during the COVID-19 pandemic. The study revealed a considerable increase in the proportions of participants consuming homemade foods (93.7%) during the pandemic (*p* = 0.003) and a significant decline in consuming fast food (9.6%) and restaurant food (5.7%) (*p*<0.001). However, healthy restaurant food consumption (31.6%) was higher during the pandemic among the participants. The proportion of participants who took four or more meals daily increased from 6.3% to 19.2% during the COVID-19 pandemic (*p*<0.001), while the percentage of participants eating breakfast increased from 76.3% to 80.8% (*p* = 0.023). Additionally, the skipping meals of the participants decreased from 34.5% pre-pandemic pandemic to 30.6% during. The reason behind skipping meals among participants was mainly due to lack of time (10.4%) before the pandemic, while it was due to trying to lose weight (9.6%) and lack of appetite (9%) during the pandemic. In case of water intake, the percentage of drinking water more than 2 liters increased from 27.8% to 36.5% during the pandemic (*p*<0.001).

**Table 2:** Eating habits pre- and during the COVID-19 pandemic.

Variables	Sub-categories	Pre-COVID-19 n (%)		During COVID-19 n (%)		*p*-value^†^
**Most consumed meals**	Homemade	435	(88.8)	459	(93.7)	0.003
	Frozen ready-to-eat meals	70	(14.3)	67	(13.7)	0.749
	Fast food	107	(21.8)	47	(9.6)	<0.001
	Restaurants	118	(24.1)	28	(5.7)	<0.001
	Healthy restaurants	118	(24.1)	155	(31.6)	<0.001
**Number of meals per day**	1–2 meals	106	(21.6)	81	(16.5)	<0.001
	3–4 meals	353	(72.0)	315	(64.3)	
	≥4 meals	31	(6.3)	94	(19.2)	
**Eating breakfast on most days**	Yes	374	(76.3)	396	(80.8)	0.023
	No	116	(23.7)	94	(19.2)	
**Skipping meals**	Yes	169	(34.5)	150	(30.6)	0.087
	No	321	(65.5)	340	(69.4)	
**Reasons for skipping meals**	To reduce food intake	30	(6.1)	33	(6.7)	0.070
	Lack of time	51	(10.4)	39	(8.0)	
	To lose weight	42	(8.6)	47	(9.6)	
	Lack of appetite	36	(7.3)	44	(9.0)	
	Fasting	1	(0.2)	8	(1.6)	
	Not applicable	330	(67.3)	319	(65.1)	
**Amount of water consumed**	<1 liter	73	(14.9)	59	(12.0)	<0.001
	1–2 liters	281	(57.3)	252	(51.4)	
	>2 liters	136	(27.8)	179	(36.5)	

*Note*: ^**†**^McNemar test

Table 3 shows the consumption of particular food products by the study participants during the COVID-19 pandemic. 8.2% did not eat fruits daily, 3.1% did not eat vegetables daily, 11.2% did not consume milk and dairy products, 3.1% did not consume meat/ chicken/ fish, and 19.4% did not consume sweets and desserts daily. However, one-third participants consumed fruits (35.1%), vegetables (37.6%), milk and milk products (39.4%) and coffee/ tea (37.3%) at least once per day. Sweet drinks such as soft drinks, canned juice, etc., were consumed less frequently among participants, 28.8% reported not consuming daily and 54.1% reported never consuming energy drinks during the pandemic.

**Table 3:** The frequency of consumption of particular foods during the COVID-19 pandemic (the study periods).

Food items	Never		1–4 times/ week		Once/ day		2–3 times/ day		≥4 times/ day	
	n	(%)	n	(%)	n	(%)	n	(%)	n	(%)
**Fruits**	40	(8.2)	205	(41.8)	172	(35.1)	58	(11.8)	15	(3.1)
**Vegetables**	15	(3.1)	152	(31.0)	184	(37.6)	128	(26.1)	11	(2.2)
**Milk and milk products**	55	(11.2)	182	(37.1)	193	(39.4)	44	(9.0)	16	(3.3)
**Meat/ chicken/ fish**	15	(3.1)	190	(38.8)	155	(31.6)	122	(24.9)	8	(1.6)
**Bread/ rice/ noodles/ pasta**	29	(5.9)	111	(22.7)	109	(22.2)	219	(44.7)	22	(4.5)
**Sweets/ desserts**	95	(19.4)	265	(54.1)	107	(21.8)	15	(3.1)	8	(1.6)
**Coffee/ tea**	62	(12.7)	138	(28.2)	183	(37.3)	74	(15.1)	33	(6.7)
**Sweet drinks (soft drinks, canned juice, etc.)**	141	(28.8)	255	(52.0)	72	(14.7)	19	(3.9)	3	(0.6)
**Energy drinks**	265	(54.1)	160	(32.7)	48	(9.8)	13	(2.7)	4	(0.8)

From the PCA, a total of two components were extracted based on eigenvalue (at least 1) and scree plot. These two components explained approximately 52% of the variance in eating habits and were labeled based on the interpretation of the factor loadings. The first factor explained 36% of the variation and the second factor explained 16% of the eating variation. From Table 4, it was observed that sweets, sweets drinks, and energy drinks loaded highly with the first factor and were labeled “Free sugars diet”. The second component is loaded positively with fruits, vegetables, milk, meat, and rice/ bread. Therefore, it was labeled a “Traditional-type diet”. A KMO of 0.78 was obtained, which is considered substantial.

**Table 4:** Component loading for the two major dietary patterns of the participants during the COVID-19 pandemic (the study periods).

Food groups	Free sugars	Traditional
Fruits	0.244	**0.651**
Vegetables	0.037	**0.745**
Milk	0.425	**0.538**
Meat	0.161	**0.668**
Bread/ rice/ noodles/ pasta	0.019	**0.668**
Sweets/ desserts	**0.730**	0.264
Coffee/ tea	0.435	0.277
Sweet drinks	**0.824**	0.051
Energy drinks	**0.846**	-0.005
**KMO**	**0.78**	

*Note*: KMO: Kaiser-Meyer-Olkin (KMO) test. The unique characteristics of each component is presented in bold. Loadings ≥0.50

### Stress and irritability

Table 5 represents stress and irritability-related information among participants before and during the pandemic by all four stress parameters. The results indicate a significant rise of physical exhaustion from 84.1% to 89.6%, emotional exhaustion from 81.2% to 90.4%, irritability from 82% to 89.6%, and tension from 84.1% to 90.6% during the pandemic compared to pre-pandemic (all *p*<0.001). The proportion of tobacco smoking among participants was slightly higher during the COVID-19 pandemic compared to the pre-COVID-19 periods (12.9% vs. 12.7%).

**Table 5:** Stress and irritability pre- and during the COVID-19 pandemic.

Variables	Sub-categories	Pre-COVID-19 n (%)		During COVID-19 n (%)		*p*-value^†^
**Physically exhausted**	A large part of the time	31	(6.3)	42	(8.6)	<0.001
	Part of the time	240	(49.0)	261	(53.3)	
	A small part of the time	141	(28.8)	136	(27.8)	
	Not at all	78	(15.9)	51	(10.4)	
**Emotionally exhausted**	A large part of the time	40	(8.2)	77	(15.7)	<0.001
	Part of the time	224	(45.7)	249	(50.8)	
	A small part of the time	134	(27.3)	117	(23.9)	
	Not at all	92	(18.8)	47	(9.6)	
**Irritable**	A large part of the time	51	(10.4)	87	(17.8)	<0.001
	Part of the time	198	(40.4)	215	(43.9)	
	A small part of the time	153	(31.2)	137	(28.0)	
	Not at all	88	(18.0)	51	(10.4)	
**Tense**	A large part of the time	66	(13.5)	105	(21.4)	<0.001
	Part of the time	226	(46.1)	198	(40.4)	
	A small part of the time	120	(24.5)	141	(28.8)	
	Not at all	78	(15.9)	46	(9.4)	

*Note*: Copenhagen Psychosocial Questionnaire (COPSOQ-II). ^**†**^McNemar test

### Physical activity

Table 6 shows that 58.2% reported not being involved in physical activity before the pandemic which decreased to 48.8% during the pandemic, and the percentage of taking physical activity more than three times per week increased from 8.2% to 13.5% (*p*<0.001).

**Table 6:** Physical activities and daily activities pre- and during the COVID-19 pandemic.

Variables	Sub-categories	Pre-COVID-19 n (%)		During COVID-19 n (%)		*p*-value^†^
**Physical activity**	No	285	(58.2)	239	(48.8)	<0.001
	1–3 times/ week	165	(33.7)	185	(37.8)	
	>3 times/ week	40	(8.2)	66	(13.5)	
**Doing household chores**	Never	130	(26.5)	115	(23.5)	<0.001
	1–3 times/ week	178	(36.3)	135	(27.6)	
	4–5 times/ week	52	(10.6)	91	(18.6)	
	Everyday	130	(26.5)	149	(30.4)	
**Screen time for entertainment**	Less than 30 minutes	91	(18.6)	51	(10.4)	<0.001
	1–2 hours	201	(41.0)	129	(26.3)	
	3–5 hours	138	(28.2)	176	(35.9)	
	More than 5 hours	60	(12.2)	134	(27.3)	
**Screen time for study or work during COVID-19 pandemic**	None	88	(18.0)	-	-	-
	1–2 hours	160	(32.7)	-	-	
	3–5 hours	123	(25.1)	-	-	
	More than 5 hours	119	(24.3)	-	-	

Note

^**†**^McNemar test

Similarly, regular household activities drastically soared from 26.5% to 30.4% during the pandemic (*p*<0.001). Spending over five hours on screen time for entertainment increased from 12.2% before the pandemic to 27.3% during the pandemic (*p*<0.001). The percentage of participants’ screen time for more than five hours for study or work pre-COVID-19 was estimated at 24.3%.

### Sleep

Table 7 shows that throughout the pandemic, the proportion of the percipients sleeping fewer than seven hours fell from 38.2% to 22% while participants sleeping more than nine hours rose from 8.2% to 22.4% (*p*<0.001). Nonetheless, a larger proportion of participants (13.1%) reported poor sleep quality during the pandemic compared to the pre-pandemic (6.1%) (*p*<0.001).

**Table 7:** Sleep pre- and during the COVID-19 pandemic.

Variables	Sub-categories	Pre-COVID-19 n (%)		During COVID-19 n (%)		*p*-value^†^
**Hours of sleep per night**	< 7 hours	187	(38.2)	108	(22.0)	<0.001
	7–9 hours	263	(53.7)	272	(55.5)	
	>9 hours	40	(8.2)	110	(22.4)	
**How would you rate your sleep quality**	Very good	148	(30.2)	167	(34.1)	<0.001
	Good	293	(59.8)	237	(48.4)	
	Poor	30	(6.1)	64	(13.1)	
	Very poor	19	(3.9)	22	(4.5)	
**Did you experience any of the following**	Slept badly and restlessly	50	(10.5)	61	(12.9)	<0.001
	Hard to go to sleep	52	(10.9)	93	(19.6)	
	Woken up too early and not been able to get back to sleep	49	(10.3)	33	(7.0)	
	Woken up several times and found it difficult to get back to sleep	26	(5.5)	38	(8.0)	
	None of the above	298	(62.7)	249	(52.5)	
**Describe your energy level**	Energized	159	(32.4)	101	(20.6)	<0.001
	Neutral	272	(55.5)	210	(42.9)	
	Lazy	59	(12.0)	179	(36.5)	

Note

^**†**^McNemar test

Sleep disturbances were also found common among the participants during the pandemic (47.5%) as opposed to pre-pandemic (37.2%). Accordingly, 20.6% of the participants reported less energized and 36.5% were lazy compared to prior time, during the shutdown (*p*<0.001).

Table 8 displays the Chi-square analysis of weight and behavioral factors by sex and residence. There was no association between weight gain and sex but participants who resided in urban areas reported a higher weight gain (48.1% vs. 37.2%, *p =* 0.014).

**Table 8:** Lifestyle changes during COVID-19 pandemic by sex and residence.

Variables	Categories	Total *n* = 490	Sex			Residence		
			Male *n* = 248	Female *n* = 242	*p*-value^†^	Urban *n* = 291	Rural *n* = 199	*p*-value^†^
**Weight** ***n* (%)**	Increased	214 (43.7)	116 (46.8)	98 (40.5)	0.307	140 (48.1)	74 (37.2)	0.014
	Same as before	215 (43.9)	105 (42.3)	110 (45.5)		112 (38.5)	103 (51.8)	
	Decreased	61(12.4)	27 (10.9)	34 (14.0)		39 (13.4)	22 (11.1)	
**Meals per day *n* (%)**	Increased	115 (23.5)	48 (19.4)	67 (27.7)	0.043	63 (21.6)	52 (26.1)	0.409
	Same as before	345 (70.4)	181 (73.0)	164 (67.8)		208 (71.5)	137 (68.8)	
	Decreased	30 (6.1)	19 (7.7)	11 (4.5)		20 (6.9)	10 (5.0)	
**Physical activity** ***n* (%)**	Increased	113 (23.1)	49 (19.8)	64 (26.4)	0.031	67 (23.0)	46 (23.1)	0.105
	Same as before	337 (68.8)	172 (69.4)	165 (68.2)		194 (66.7)	143 (71.9)	
	Decreased	40 (8.2)	27 (10.9)	13 (5.4)		30 (10.3)	10 (5.0)	
**Screen time (entertainment) *n* (%)**	Increased	205 (41.8)	105 (42.3)	100 (41.3)	0.721	121 (41.6)	84 (42.2)	0.069
	Same as before	257 (52.4)	127 (51.2)	130 (53.7)		159 (54.6)	98 (49.2)	
	Decreased	28 (5.7)	16 (6.5)	12 (5.0)		11 (3.8)	17 (8.5)	
**Sleep (h)** ***n* (%)**	Increased	162 (33.1)	90 (36.3)	72 (29.8)	0.306	102 (35.1)	60 (30.2)	0.484
	Same as before	307 (62.7)	148 (59.7)	159 (65.7)		176 (60.5)	131 (65.8)	
	Decreased	21 (4.3)	10 (4.0)	11 (4.5)		13 (4.5)	8 (4.0)	
**Sleep disturbances** ***n* (%)**	Increased	64 (13.1)	36 (14.5)	28 (11.6)	0.392	38 (13.1)	26 (13.1)	0.999
	Same as before	350 (71.4)	178 (71.8)	172 (71.1)		208 (71.5)	142 (71.4)	
	Decreased	76 (15.5)	34 (13.7)	42 (17.4)		45 (15.5)	31 (15.6)	

Note

^**†**^Chi-square test

Significantly more women than men reported eating more meals each day (27.7% vs. 19.4%, *p* = 0.043), and doing exercise (26.4% vs. 19.8%, *p* = 0.031) reported. There was no relationship between sleep issues, screen time and sex, or residence (Table 8).

## Discussion

This study gives a picture of the dietary patterns, physical activity levels, and lifestyle modifications of Bangladeshi adolescents who participated in the survey between December 2020 and May 2021 during the COVID-19 pandemic. To the best of our knowledge, this is the first study investigating how the COVID-19 lockdown affected eating behaviors, physical activity levels, and lifestyle changes.

According to the findings, nearly half of the respondents (43.7%) reported weight gain as a result of lockdown measures taken during the pandemic, and higher than one-third of participants (36.7%) reported raised hunger and satiety, which is consistent with a study carried out in the United Arab Emirates in the UAE [1]. Comparable findings from the United States, Italy, France, and Kuwait also indicated that the surveyed population gained more weight during the pandemic [33–36]. Similar to the survey conducted among UAE citizens [1], the current study found that most of the study participants (73.9%) reported satisfactory health status during the lockdown. Since, until now COVID-19 has no permanent therapies, healthy eating habits are considered beneficial, and elective micronutrients might provide an advantage, particularly for vulnerable populations [36]. During the lockdown, most participants consumed homemade foods (93.7%) compared to before the pandemic which showed similarity with previous studies [1, 37]. However, due to adolescents’ proficiency with food delivery services, the proportion of fast food meals consumed by them remained stable. In addition, fruits and vegetables are considered as an important source of fiber, vitamins, minerals, and antioxidants which can provide better health status with a lower risk of chronic diseases, as well as improved immunity [38–40]. According to the study, half of the adolescents (50%) did not consume fruits, 48.3% did not consume dairy products, and 41.9% did not consume protein daily. 59.1% consumed coffee/ tea at least once per day which showed consistency with a previous study [1], though the results regarding vegetable consumption do not agree with the present study. Another study conducted in the Middle East and North Africa region reported that 57% of participants prepared their own meals and 79% ate less often at restaurants which also showed consistency with our study [41] and an Italian survey reported a large decrease in the consumption of takeout food, especially in families with children between the ages of 5 and 11, as a result of the complete shutdown [37].

The present study reported tobacco use among adolescents increased slightly during the pandemic. In contrast, a study related to COVID-19 performed among the people of Italy during the COVID-19 pandemic revealed a decreased level of smoking among the study participants [36], which might be due to the fear induced in smokers of the increased risk of respiratory disease and deadly consequence of COVID-19 [42].

Many mental health issues, such as anxiety, stress, anger, despair, and frustration, gradually surfaced during the start of the COVID-19 epidemic [43, 44]. In this study, exhaustion, irritability, and tension increased significantly during the pandemic in contrast to before which is similar to a prior study [1], and could have a long-term impact on the mental well-being of the people [45]. Additionally, the burden of mental health disorders is not a new problem in Bangladesh, but unfortunately is a largely unrecognized and under-researched area [46], which needs more emphasis for the healthy mental status of every individual. In addition, a prior study indicated that stress and anxiety could affect sleep quality and energy levels [1]. The present research also reported poor sleep quality, less energy, and increased laziness during the pandemic compared to pre-pandemic. A systematic review and a meta-analysis conducted among 54,231 participants from 13 countries during the pandemic reported that sleep problems among all populations were 35.7%, where COVID patients appeared to be the most affected groups including healthcare workers (36%) and general populations (32%) [47]. In this case, the use of telehealth services has been found beneficial to support patients in delivering mental health services [48].

This study revealed that nearly half of the participants (48.8%) stated not involved in any kind of physical activity during the lockdown, which is slightly more than the previous study conducted in UAE [1]. This finding is also similar to another study conducted during the pandemic in Bangladesh [49], as well as in other countries [50–52]. The influences of home quarantine at the time of the pandemic brought significant changes in lifestyle behavior and sedentary behavior [50]. Our study reported more people used screens for amusement for longer than five hours during the pandemic than they did before, which is consistent with a previous study conducted in the UAE [1]. Reduced physical activity has the potential to affect weight as well as future NCD risk [53, 54].

### Limitations

The study had several limitations. The results may have potential biases due to the study’s self-reported questionnaire. Though the questionnaire was adopted from previous literature [1, 25–27], the questionnaire was not validated in the context of Bangladesh. Further study with validated instruments would be more appropriate in this regard. The study was cross-sectional in nature and it cannot determine casualty. Due to the small sample size and convenient sampling technique, the study cannot be regarded as a representative of adolescents in Bangladesh or other countries. Moreover, the selection bias was acknowledged due to the online survey. Thus, a further study with a larger sample including a random sampling technique would be needed to overcome these limitations.

## Conclusions

According to this study’s findings, the COVID-19 pandemic in Bangladesh resulted in unfavorable lifestyle changes including unhealthy food choices, insufficient physical activity, increased screen time, and psychological issues for adolescents. The findings suggest to promote healthy lifestyle among adolescents during the emergencies like COVID-19 pandemic among adolescents targeting balanced diet, regular physical exercising, and controlling secondary screen time. To address the psychological issues, virtual awareness sessions can be initiated and encouraged to strengthen the psychosocial support through close networks and peers.

## Supporting information

S1 File (XLSX)
